# Charles Darwin – The 200th Anniversary 

**Published:** 2009-04-25

**Authors:** Popa Florian, Craciunas Sorin, Vlad Ciurea Alexandru, Lorin Purcarea Victor

**Affiliations:** * „Carol Davila” University of Medicine and Pharmacy, Bucharest, Romania; ** First Neurosurgical Clinic „Bagdasar Arseni” Clinical Emergency Hospital, Bucharest, Romania

Every age in the history of civilized men was dominated by a specific set of ideas or ideologies. However, Darwin's *On the Origin of Species* (1859) must be certainly mentioned as the top of the list.

The year 2009 marks the 200th anniversary of Darwin’s birth and the sesquicentennial of the *Origin*’s publication. Scientists, universities, natural history museums, and other sorts of people, various institutions all over the world are celebrating this anniversary by organizing scientific lectures, symposia, exhibitions.

Charles Robert Darwin, the scientist, had produced a fertile collection of work during his lifetime. Of his 19 books, his most prominent and remarkable work, *On the Origin of Species*, was published in 1859 when he was exactly 50 years old. There have been many theories concerning the reason why he waited so long to publish his thoughts on the origin of species, after he arrived from his voyage in 1836. 

Darwin was born in Shrewsbury, England, on February 12, 1809, in the same day with Abraham Lincoln. His father, Robert Darwin, was a rich physician with one of the most extended medical practices outside London. His paternal grandfather, Erasmus Darwin, was both a physician and a famous nature novelist. As a young boy, Darwin showed an interest in natural history but started his superior schooling in Medicine, in Edinburgh, a science he shortly learned to dislike. Later, at Cambridge, where he went to arrange for a profession in the clergy, he showed no interest in his theological studies, but became familiar with a botany professor, the Reverend John Henslow, who was to become his mentor and to have a thoughtful effect on his life. It was Henslow who encouraged Darwin to take an extended sea journey and exploration of the world outside of England, after his graduation from Cambridge.

Darwin was primarily a naturalist. His preferred method was that of the naturalist. He made a series of observations and developed an inference from this fact. He considered this approach to be the inductive technique. He recorded the fact that he considered himself a true adept of Bacon in his autobiography. Darwin was a very enthusiastic observer, and there is no doubt that observation was his most prolific approach. However, he was also a skilled experimentalist and he led numerous experiments mainly in his botanical researches.

**Figure:**
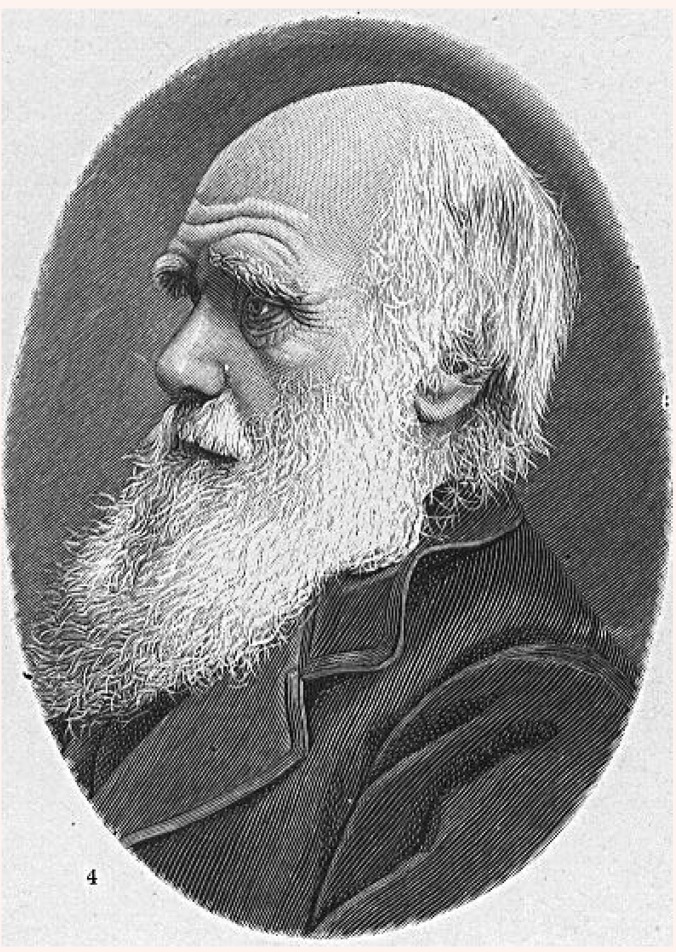


The most extensively used technique in the Physical Sciences is the experiment. However, in his evolutionary studies, Darwin had to cope with an issue that is irrelevant in most of the Physical Sciences except for Geology and Cosmology, the time factor. One cannot make an experiment with biological actions in the past. Phenomena like the disappearance of the dinosaurs and all other evolutionary events are unapproachable to the experimental method and involve a completely different methodology, that of the so-called „historical narratives.” One develops an unreal scenario of the past events in this method, based on their consequences. Darwin used this method very successfully in his biogeographically reconstructions.

A strict approval of every word in the Bible was the usual view of every Christian. In addition, God created everything we see in this world. Natural Theology added the trust that, at the time of creation God had also established a set of rules that would maintain the ideal adaptation of a well-confined world. Darwin defied all three major pieces of this belief. First, he affirmed that the world is developing rather than remaining invariable; secondly, that new species are not particularly created but derived from common ancestors; and thirdly, that the adaptation of each species is constantly regulated by the course of natural selection. In Darwin's theories, there is no necessity for divine intrusion or the action of supernatural forces in the whole process of the evolution of the living world. Thus, Darwin's revolutionary idea was to substitute the divinely controlled world with a strictly material world, managed according to the natural laws.

Darwin's most innovative and most important new idea was that of natural selection. Why were the people so hostile to this theory for such a long time? It is clear that the theoretical support of the period and particularly the almost universal approval of typological thinking- what Popper called essentialism-was involved. This kind of viewpoints were first introduced into philosophy by Plato and the Pythagoreans, who hypothesized that the world consisted of a finite number of classes of things and that only the type (core) of each of these classes of objects had reality, all the seeming variations of these types being unimportant. Darwin rejected such an explanation for organic diversity. As an alternative, he introduced the philosophy we now refer to as population thinking. By developing the concept of bio-populations, Darwin made a fundamental contribution to modern thinking, even though he himself was not always constant in its acceptance. 

When Immanuel Kant, attempted to develop a philosophy of biology on the foundation of Newton’s physical viewpoint, he failed. Finally, he assumed that Biology is different from the Physical Sciences, and that we must find some philosophical aspect that was not used by Newton. 

Indeed, he considered he had found such a thing in Aristotle's fourth cause, the final cause. This had a rather unhelpful effect on the German nineteenth- century thinking, because an unproven trust on teleology played a central role in the philosophies of all of Kant's supporters. It was Darwin's immense realization of being able to elucidate all the phenomena for which Kant had considered he needed to invoke teleology, by natural selection. The simple automatic process of natural selection, creating plentiful variation in every generation and always removing the weak individuals, can elucidate all processes and phenomena that, prior to 1859, could be explained only by teleology. Nowadays we still recognize four teleological phenomena or processes in nature, but they can all be explained by the laws of Chemistry and Physics, while teleology, such as that assumed by Kant, does not exist.

Determinism was a dominant philosophy prior to Darwin. As Laplace affirmed that, if he knew the exact location and motion of every object in the universe, then he would have been able to calculate every detail of the future history of the world. There was no room in his thinking for probability. Darwin also paid strict lip service to such determinism. He accepted the usual belief of his period that every chance process in the universe had a cause. 

Ideas of science in the Newtonian thinking were usually based on laws. However, Darwin accepted this vision. Therefore, we find that he used the expression "law" very generously in the Origin. He called any cause or event that seemed to occur regularly a law. The present widely adopted point of view is that ideas in evolutionary biology are fundamented on concepts rather than laws, and this subdivision of science certainly has copious concepts which to base theories on.

To summarize Darwin's contributions to the thinking of modern men: He was responsible for the substitution of a world vision based on Christian doctrine by a strictly secular worldview. Furthermore, his writings led to the rejection of some previously leading worldviews such as essentialism, finalism, determinism, and the suitability of Newtonian laws for the elucidation of evolution. He replaced these refuted ideas with a number of new ones of wide- reaching significance, also outside of Biology, such as biopopulation, natural selection, the significance of chance and contingency, the explanatory importance of the time factor (historical narratives), and the importance of the social group for the origin of ethics. Almost every constituent in the system of modern man's thinking is somehow affected by one or another of Darwin's theoretical contributions. His opus as a whole is the base of a rapidly developing new thinking of Biology.

Darwin's philosophy had a deep impact also outside the field of natural sciences. Applied to politics it led to the talk about "favored races" and the dogma that nations fight so that the fittest shall survive. Darwin himself once said: "Believing, as I do, that man in the distant future will be a more perfect creature than he is now and that he and all other sentient beings are doomed to complete annihilation after such long-continued slow progress, is an intolerable thought. To those who freely admit the immortality of the human soul, the destruction of our world will not appear so dreadful." 

At Darwin’s 200th celebration, it is about time that public schools taught the theory of evolution by natural selection for what it is: one of the greatest intellectual achievements of all times. It completed the Copernican Revolution and brought the scientific revolution to mankind, the methodological promise to elucidate all of nature, lifeless as well as living, as matter in movement governed by natural laws.
